# NATURAL HISTORY OF THE GASTROJEJUNAL ANASTOMOSIS IN PATIENTS UNDERGOING ROUX-EN-Y GASTRIC BYPASS

**DOI:** 10.1590/S0004-2803.24612025-065

**Published:** 2026-03-02

**Authors:** Gustavo Henrique Xavier CASEIRO, Vitor Ottoboni BRUNALDI, Maisa BUISSA, Roberto Luiz KAISER-JUNIOR, Carolina Colombelli PACCA, Luiz Gustavo de QUADROS

**Affiliations:** 1 São José do Rio Preto Medical School - FAMERP, São José do Rio Preto, SP, Brazil.; 2 Kaiser Clinic, São José do Rio Preto, SP, Brazil.; 3 University of Sao Paulo, Ribeirão Preto Medical School, Surgery and Anatomy Department, Center for Digestive Endoscopy, Ribeirão Preto,SP, Brazil.; 4 Mayo Clinic, Gastroenterology and Hepatology division, Rochester, USA.; 5 São Paulo State University (Unesp), Institute of Biosciences, Letters and Exact Sciences (IBILCE), Postgraduate Program in Microbiology, São José do Rio Preto, SP, Brazil.

**Keywords:** Bariatric surgery, endoscopy, obesity, Bypass gástrico, cirurgia bariátrica, endoscopia bariátrica, anastomose gastrojejunal

## Abstract

**Background:**

Obesity is considered a global epidemic and has shown a progressive increase in recent decades. Bariatric surgery, such as Roux-en-Y gastric bypass (RYGB), is the most effective sustainable weight loss option. However, weight regain is one of the challenges facing patients and is attributed to several factors, including dilatation of the gastrojejunal anastomosis (GJA).

**Objectives:**

The central objective of this study is to correlate the influence of time on GJA widening in patients undergoing RYGB over a 7-year period.

**Methods:**

Prospective and longitudinal study conducted over 7 years in patients undergoing RYGB. Surgical and endoscopic procedures were performed in a medium hospital in the same city. Weight, body mass index (BMI) and GJA size were assessed at intervals of 2, 6, 12, 24, 30, 48, 60, and 72 months after the surgical procedure.

**Results:**

The highest mean value in the distribution of anastomosis diameter was 20±2.27 mm at 72 months after surgery. The lowest mean value was 10.2±1.70 mm at 2 months after surgery. The analysis showed that there is a significant effect of time on anastomosis diameter, with statistically significant differences in the mean value between 2, 6, 12, 24, 60, and 72 months after surgery (F(1,724,5,172)=9.555, *P*<0.05).

**Conclusion:**

Multiple comparative analysis showed that there are statistically significant differences between the mean lengths of anastomosis across the times studied, with a greater influence of the time factor 24 months after surgery.

## HIGHLIGHTS

• First long-term prospective study tracking gastrojejunal anastomosis (GJA) dilation over 7 years post-RYGB.

• GJA diameter increased significantly from 10.2±1.70 mm (2 months) to 20±2.27 mm (72 months).

• Critical timepoint: 24 months post-surgery marked a 55% enlargement in anastomosis diameter (P<0.05).

• Although GJA dilation increased significantly over time, no significant correlation was found with weight regain.

## RESUMO

**Contexto:**

A obesidade é considerada uma epidemia global e tem apresentado aumento progressivo nas últimas décadas. A cirurgia bariátrica, como o bypass gástrico em Y de Roux (RYGB), é a opção mais eficaz para perda de peso sustentável. No entanto, o reganho de peso é um dos desafios enfrentados pelos pacientes e é atribuído a vários fatores, incluindo a dilatação da anastomose gastrojejunal (GJA).

**Objetivos:**

O objetivo deste estudo foi correlacionar a influência do tempo no alargamento da GJA em pacientes submetidos a RYGB ao longo de um período de 7 anos.

**Métodos:**

Estudo prospectivo e longitudinal realizado ao longo de 7 anos em pacientes submetidos a RYGB. Os procedimentos cirúrgicos e endoscópicos foram realizados em um hospital de médio porte na mesma cidade. Peso, índice de massa corporal (IMC) e tamanho da GJA foram avaliados em intervalos de 2, 6, 12, 24, 30, 48, 60 e 72 meses após o procedimento cirúrgico.

**Resultados:**

O maior valor médio na distribuição do diâmetro da anastomose foi de 20±2,27 mm aos 72 meses após a cirurgia. O menor valor médio foi de 10,2±1,70 mm aos 2 meses após a cirurgia. A análise mostrou que há um efeito significativo do tempo no diâmetro da anastomose, com diferenças estatisticamente significativas no valor médio entre 2, 6, 12, 24, 60 e 72 meses após a cirurgia (F(1,724,5,172)=9,555, *P*<0,05).

**Conclusão:**

A análise comparativa múltipla mostrou que há diferenças estatisticamente significativas entre os comprimentos médios das anastomoses ao longo dos tempos estudados, com maior influência do fator tempo 24 meses após a cirurgia.

## INTRODUCTION

RYGB is an effective and safe technique for the treatment of morbid obesity and is constantly evolving and improving^1^ ^-^ ^3^. Several technical aspects of RYGB remain under discussion, such as the adequate size of the gastric pouch, the length of the biliopancreatic loop, the use of staple line reinforcement, and especially, the ideal size of gastrojejunal anastomosis (GJA)^4^ ^-^ ^6^. These discussions consider both the success of treatment and the risk of insufficient weight loss or weight regain^7^ ^,^ ^8^.

Weight regain is typically observed two to seven years after RYGB surgery^9^. Although it often results from a combination of factors, the link between gastrojejunal anastomosis (GJA) dilatation and abnormal weight gain has been widely studied. The main reason for this relationship is believed to be the loss of the limiting property of gastric reservoir emptying^10^ ^-^ ^12^.

The increased diameter of the GJA has been associated with lower success rates in weight control, with the hypothesis that a larger diameter anastomosis would facilitate the rapid emptying of the gastric reservoir, reducing the patient’s satiety^11^ ^-^ ^14^. Although RYGB and GJA are well studied, there are currently insufficient studies that detail the real influence of time on the diameter of the anastomosis.

Upper digestive endoscopy (UDE) is an important investigative tool for diagnosing gastrointestinal diseases and assessing the size of the GJA before and after RYGB^10^ ^,^ ^15^. Preoperative UDE, even in asymptomatic patients, guides the treatment of modifiable conditions prior to bariatric surgery. Endoscopic evaluation may also lead to identifying anatomical abnormalities that can be treated during surgery, assist in the choice of surgical approach, or guide the decision not to proceed with the surgical procedure altogether. Additionally, UDE is widely indicated during postoperative follow-up of RYGB to diagnose and treat complications, as well as to evaluate possible factors associated with weight loss failure^16^ ^,^ ^17^.

This study aims to analyze the evolution of GJA diameter measurements and their possible correlations with surgical outcomes in patients undergoing RYGB, prospectively and over the long term.

## METHODS

### Study design and setting

A prospective longitudinal study was conducted over seven years in patients who were initially candidates for surgical treatment of obesity using the RYGB technique.

The patients were consecutively selected after RYGB indication and before preoperative UDE was performed. All study participants gave their informed consent. The selection and monitoring of participants were carried out in a private day hospital in the countryside of the State of São Paulo, Brazil. Surgical procedures were performed in a medium-sized hospital in the same city. The study was approved by the Ethics Committee (CAAE 25405313.3.0000.5629) and was registered as a clinical trial in the US Good Clinical Practice (#NCT03106207).

### Study population

Adult patients over 18 years of age with a previous clinical indication for surgical treatment of obesity were included in the study when they agreed to participate and comply with all the follow-up proposed. Participants were excluded if they experienced pregnancy during the study period, had psychiatric illness or inability to understand the nature of the investigation, uncontrolled comorbid conditions, refused to undergo esophagogastroduodenoscopy, or were unable to complete follow-up.

In total, 50 participants were included in the study from January to May 2014. For each patient, preoperative digestive endoscopy, RYGB surgery, intraoperative endoscopy, and postoperative digestive endoscopy were performed at different intervals throughout the study, always by the same medical team. Data were also collected during the procedures, including length measurements along the small curvature of the gastric pouch, GJA measurements of the maximum diameter, assessment of jejunal loops for mucosal lesions, and angulation of the afferent loop.

### Upper digestive endoscopy

Preoperative UDE was performed after indication of surgery for obesity treatment by the multidisciplinary team (surgeon, nutritionist, psychologist, physiotherapist, endocrinologist, cardiologist, and anesthesiologist). Endoscopic procedures (preoperative, intraoperative, and postoperative) were performed by a team of certified endoscopists with experience in bariatric endoscopy. The procedures were performed with the patient in the left lateral decubitus position, using cardiorespiratory monitoring, supplemental oxygen, and peripheral venous access. For sedation, propofol and fentanyl citrate were administered by an anesthesiologist.

Intraoperative endoscopy was performed with an Olympus CV 160 endoscope (Tokyo, Japan) to assess the size of the GJA and visualize possible suture line flaws. Endoscopy was performed with minimal insufflation using a CO2 pump (Olympus, Tokyo, Japan); findings were considered satisfactory when the device passed through the anastomosis without leakage findings and with the GJA measuring 10mm in diameter. Postoperative endoscopy was performed 2, 6, 12, 24, 30, 48, 60, and 72 months after RYGB in an outpatient setting by the same medical team. An EVIS EXERA II processor with CV 180 gastroscopes (Olympus Medical Inc., Tokyo, Japan) was used to perform the examinations.

The following parameters were assessed during the endoscopic examination: mucosa of the esophageal and gastric pouch, measurements of the lengths along the small curvature of the gastric pouch, jejunal loops for mucosal lesions and angulation of the afferent loop, and GJA measurements at its maximum diameter. The measurement was performed with a scientifically validated graduated endoscopic ruler^18^.

### Technique of laparoscopic Roux-en-Y gastric bypass

All RYGBs were performed by a leading surgeon with more than ten years of experience in surgical treatment of obesity (>7,000 surgeries) and laparoscopy. The procedure was performed using a laparoscopic approach with five trocars. The gastric pouch was created using a 32F Fouchet-type probe (11.0 mm) for calibration and was approximately 4 cm in length. The gastrojejunal anastomosis was created at 60 cm from the Treitz angle and was calibrated to approximately 11 mm using a 32F Fouchet probe. The jejunojejunal anastomosis was performed 160 cm distal to the GJA. Both Petersen’s space and the mesenteric defect were closed to prevent internal hernias.

### Statistical analysis

Statistical analyses were performed using SPSS software version 1.4 (August 2, 2020) and R version 4.0.4 (2021-02-15, Copyright (C) 2021 - The R Foundation for Statistical Computing). Multiple definitions were applied to calculate the indicators associated with the surgical procedure. Parametric tests (Shapiro-Wilk test and Levene’s test) and ANOVA for repeated measures were performed, considering the values of the anastomosis diameter as the dependent variable and the time intervals of the study as a factor, allowing observation of the effect of time on the mean value of the anastomosis diameter.

To avoid type 1 error due to missing values during follow-up, a Greenhouse-Geisser correction was performed. Since the ANOVA test statistically demonstrated that not all anastomosis diameter means in the periods are equal, a post hoc analysis was performed using the Sidak method. Pearson’s correlation test was used to assess the level of association between the anastomosis diameter and the weight lost at 2, 6, 12, 24, 60, and 72 months.

## RESULTS

A total of 50 patients were initially recruited, but 26% (n=13) were excluded due to loss to follow-up during the study period. Therefore, 37 patients completed the study (74%) with valid information on weight, BMI, and anastomosis size at the time of surgery and after 2, 6, 12, 24, 60, and 72 months.

The age of the participants at the time of the study ranged from 18 to 55 years with a mean of 34.3±9.4 years. Regarding sex, 78.4% (n=29) were women, and the height of the participants ranged from 1.47 m to 1.85 m. The preoperative weight ranged from 89.0kg to 152.0kg, and preoperative BMI ranged from 31.2 kg/m^2^ to 49.2 kg/m^2^ (Table 1).

**TABLE 1:** Demographic characteristics of the included population.

Sociodemographic information	No. (%)
Total	%
Age, SD	34.38±9.497
Sex	
Female	29(78.4)
Male	8(21.6)
Height	1.67±0.09
Preoperative weight	116.28(±17.9)
Preoperative BMI	41.37(±4.04)

SD: standard deviation. BMI: body mass index. %: percentage.

**FIGURE 1:**
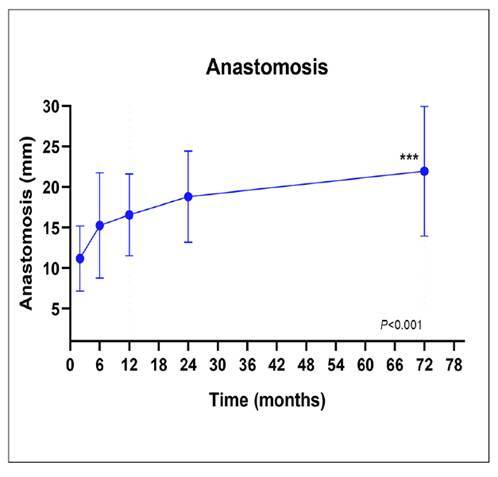
Mean diameter of the anastomosis by time after RYGB.

The mean diameter of the GJA was calculated for all time points in the study. Figure 1 demonstrates the mean diameter over time. The highest mean value was 20±2.27mm, occurring at 72 months after surgery (longest follow-up). The lowest mean value was 10.2±1.70mm assessed at 2 months after surgery. 

The analysis showed that there is an effect of time on the anastomosis, with statistically significant differences in the mean value of anastomosis length between 2, 6, 12, 24, 60, and 72 months after surgery (F(1,724, 5,172)=9.555, *P*<0.05). The statistical analysis revealed significant differences (*P*<0.05) between the mean diameter of the anastomosis at the initial surgical procedure and its mean diameter after 24 months. This disparity indicated a substantial increase of 5.5 millimeters in the anastomosis diameter at the 24-month mark, corresponding to a 55% enlargement compared to the diameter observed at the time of surgery.

Additionally, noteworthy statistical distinctions (*P*<0.05) were evident in the mean anastomosis diameter between the two-month and 72-month postoperative periods. These differences represented a substantial variance of 9.75 millimeters between the means. The diameter of the anastomosis during the 72-month interval displayed a noteworthy enhancement of 95.12% compared to the diameter noted merely two months after the surgical intervention.

**FIGURE 2:**
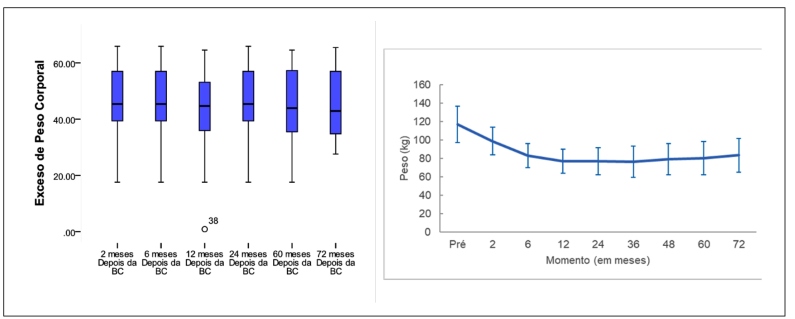
Distribution of the proportion of excess weight loss by time interval.

The evolution of anthropometric measurements was also evaluated before the surgical procedure. Among the 37 participants, the mean preoperative weight was 115.82±17.86 kg. Weight reduction was noticeable until the 12-month mark, and from 24 months onwards, it stabilized, experiencing a subsequent increase by the 72-month mark in comparison to earlier stages. After 72 months, patients (N=17) presented a mean weight of 86.26±20.86 kg. The analysis indicated an effect of time on weight, with the preoperative and 2-month measurements showing significantly higher values than the other time points (*P*<0.001). The proportion of weight lost by the participants during the study period can be observed in Figure 2.

Examining the study period’s percentage of excessive weight loss, the lowest mean occurred at 41.42±10.67% after two months post-surgery, while the highest mean reached 95.66±25.78% at 24 months. BMI calculations aligned with weight trends, showing higher values at preoperative and two-month intervals compared to others (*P*<0.001), with an increase at 24 months (*P*<0.001).

The WHO-based obesity classification pre-surgery found 5.4% grade I, 24.3% grade II, and 70.3% grade III obesity. Post-surgery, 6-month anthropometric analysis from 17 participants yielded a mean weight of 80.67±13.88 kg. BMI results showed 11.8% achieving adequate weight, 52.9% classified as overweight, and 35.3% as grade I obese. After six months, substantial weight loss led to a shift in classification to controlled obesity, exceeding 20% according to SBCBM criteria.

**FIGURE 3:**
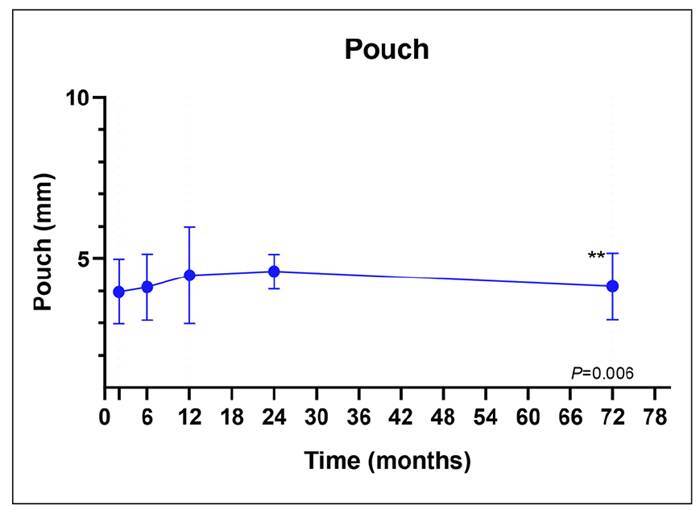
Distribution of the pouch size by time interval.

In relation to the pouch size, through the implementation of repeated measures analysis of variance, a significant alteration in pouch dimensions over the conducted assessments was identified (*P*=0.019). Specifically, the 2-month evaluation period exhibited notably smaller dimensions than those recorded at 12 months (*P*=0.025), 24 months (*P*<0.001), and 72 months (*P*=0.006). Conversely, at the 24-month mark, dimensions were significantly greater than those at the 72-month juncture (*P*=0.038). To mitigate potential type 1 error arising from multiple testing, time intervals of 36, 48, and 60 months were excluded from this analysis (Figure 3).

**FIGURE 4:**
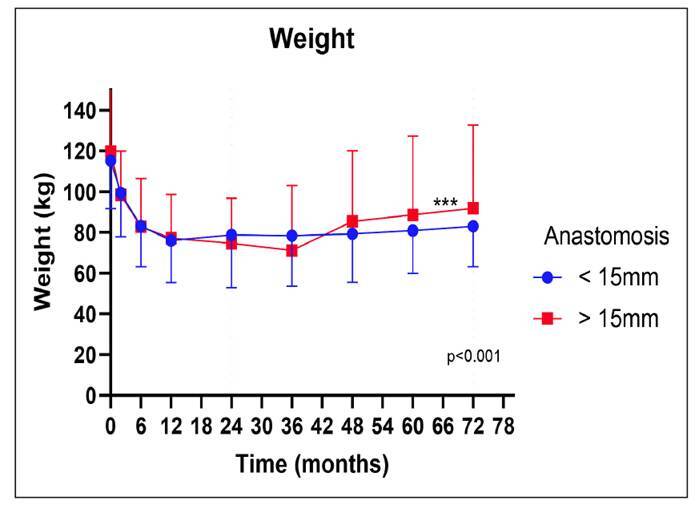
Evolution of weight according to the anastomosis group. ***Statistically significant difference (*P*<0.05).

Comparative analyses were conducted between participants with GJA diameters greater than and less than 15 mm concerning weight and BMI. Utilizing analysis of variance with repeated measures, it was determined that the anastomosis groups did not display significant differences in weight across the evaluation period (*P*=0.708). Nevertheless, a noteworthy alteration in weight during the assessment period (*P*<0.001) was evident between the two anastomosis groups (Figure 4).

## DISCUSSION

In this prospective cohort study, we tracked the evolution of the gastrojejunal anastomosis (GJA) in patients undergoing RouxenY gastric bypass (RYGB) over seven years, evaluating its potential impact on obesitytreatment outcomes. By following a single patient group with standardized procedures performed by the same surgical and endoscopic team, we were able to apply robust statistical methods across multiple time points and assess a range of clinical endpoints. Critical monitoring of anatomical changes in the surgically altered structures is essential for optimizing bariatric interventions and improving longterm patient success.

Obesity arises from multifactorial mechanisms encompassing genetic, environmental, political, psychosocial, cultural, and biological factors^19^ ^-^ ^21^. Consequently, therapeutic approaches have evolved toward greater comprehensiveness, integrating pharmacological agents, surgical procedures, and advanced endoscopic modalities-such as argon plasma coagulation and endoscopic suturing of the GJA-to enhance weightloss durability^10^ ^,^ ^13^ ^,^ ^22^ ^,^ ^23^.

Endoscopy assumes a central role throughout the bariatric care pathway. In the preoperative phase, it excludes occult comorbidities and delineates individual anatomical variations, thereby informing surgical planning^16^. During postoperative surveillance, endoscopic evaluation facilitates early identification and endoscopic management of complications, including ulcers, strictures, and leaks^16^ ^,^ ^24^.

Technically, the creation of the GJA constitutes a critical step in RYGB. Variations in anastomotic construction not only influence the incidence of major adverse events-such as bleeding, stenosis, fistula formation, and marginal ulceration-but may also affect longterm weight loss trajectories^11^ ^,^ ^12^ ^,^ ^25^.

AbuDayyeh et al. examined 165 RYGB patients who underwent upper-digestive endoscopy for various indications and demonstrated a positive correlation between GJA diameter and subsequent weight regain, defined as ≥20% regain of excess weight lost. They reported that each 10 mm increase in GJA diameter elevated the risk of weight regain by 8% at five years postRYGB^10^.

Consistent with these findings, our analysis of variance revealed a significant progressive increase in GJA diameter across followup visits (*P*<0.001), corroborating the gradual dilation reported in the literature.

Weight loss after RYGB typically accelerates in the first 24 months and may plateau or partially rebound thereafter. In our cohort, mean weight loss reached 36.55±5.68 kg at 24 months, aligning with established postoperative trajectories.

A systematic review of published studies indicates that most are retrospective, crosssectional, and lack standardized, validated tools for measuring GJA size or gastric pouch length. In contrast, we implemented sequential, quantitative endoscopic assessments within a longitudinal, longterm framework^5^ ^,^ ^18^ ^,^ ^26^ ^,^ ^27^.

Although prospective, longterm cohort studies require considerable resources and sustained patient engagement, they offer unparalleled insight into the natural history of bariatric outcomes and enable temporal sequencing of events^28^ ^,^ ^29^. We encountered expected data attrition-due to patient relocation, pregnancies, or delays in scheduled endoscopies during the COVID19 pandemic-but these factors were inherent to the study design and are transparently acknowledged.

## CONCLUSION

Our sevenyear prospective followup demonstrated a significant progressive increase in gastrojejunal anastomosis diameter, with the most pronounced widening occurring after 24 months (*P*<0.001). Although substantial weight loss was achieved by 24 months and partially maintained at 72 months, our data did not reveal a statistically significant correlation between anastomosis size and longterm weight change. These findings underscore that, despite anastomotic dilation over time, weight maintenance after RYGB is influenced by multiple factors beyond GJA diameter. Importantly, they reinforce the critical role of periodic digestive endoscopy in the longterm surveillance of RYGB patients to identify clinically relevant anastomotic changes.

## Data Availability

Data-available-upon-request

## References

[1] Jakobsen GS, Småstuen MC, Sandbu R, Nordstrand N, Hofsø D, Lindberg M (2018). Association of Bariatric Surgery vs Medical Obesity Treatment With Long-term Medical Complications and Obesity-Related Comorbidities. JAMA.

[2] English WJ, DeMaria EJ, Brethauer SA, Mattar SG, Rosenthal RJ, Morton JM (2018). American Society for Metabolic and Bariatric Surgery estimation of metabolic and bariatric procedures performed in the United States in 2016. Surg Obes Relat Dis.

[3] Kang JH, Le QA (2017). Effectiveness of bariatric surgical procedures. Medicine.

[4] Madan AK, Harper JL, Tichansky DS (2008). Techniques of laparoscopic gastric bypass: on-line survey of American Society for Bariatric Surgery practicing surgeons. Surg Obes Relat Dis.

[5] Mahawar K, Sharples AJ, Graham Y (2020). A systematic review of the effect of gastric pouch and/or gastrojejunostomy (stoma) size on weight loss outcomes with Roux-en-Y gastric bypass. Surg Endosc.

[6] Castagneto-Gissey L, Angelini G, Casella-Mariolo JR, Marini P, Mingrone G, Casella G (2020). The jejunum is the key factor in insulin resistance. Surg Obes Relat Dis.

[7] El Ansari W, Elhag W (2021). Weight Regain and Insufficient Weight Loss After Bariatric Surgery: Definitions, Prevalence, Mechanisms, Predictors, Prevention and Management Strategies, and Knowledge Gaps-a Scoping Review. Obes Surg.

[8] Iglesias-Lopez C, Obach M, Vallano A, Agustí A (2021). Comparison of regulatory pathways for the approval of advanced therapies in the European Union and the United States. Cytotherapy.

[9] Mullady DK, Lautz DB, Thompson CC (2009). Treatment of weight regain after gastric bypass surgery when using a new endoscopic platform: initial experience and early outcomes (with video). Gastrointest Endosc.

[10] Abu Dayyeh BK, Lautz DB, Thompson CC (2011). Gastrojejunal stoma diameter predicts weight regain after Roux-en-Y gastric bypass. Clin Gastroenterol Hepatol.

[11] Brunaldi VO, Jirapinyo P, de Moura DTH, Okazaki O, Bernardo WM, Galvão M (2018). Endoscopic Treatment of Weight Regain Following Roux-en-Y Gastric Bypass: a Systematic Review and Meta-analysis. Obes Surg.

[12] Ramos AC, Marchesini JC, de Souza Bastos EL, Ramos MG, de Souza MDG, Campos JM (2017). The Role of Gastrojejunostomy Size on Gastric Bypass Weight Loss. Obes Surg.

[13] Orlandini B, Gallo C, Boškoski I, Bove V, Costamagna G (2020). Procedures and devices for bariatric and metabolic endoscopy. Ther Adv Gastrointest Endosc.

[14] de Quadros LG, Neto MG, Marchesini JC, Teixeira A, Grecco E, Junior RLK (2020). Endoscopic Argon Plasma Coagulation vs. Multidisciplinary Evaluation in the Management of Weight Regain After Gastric Bypass Surgery: a Randomized Controlled Trial with SHAM Group. Obes Surg.

[15] Sharma A, Davies R, Kapoor A, Islam H, Webber L, Jayasena CN (2022). The effect of hormone replacement therapy on cognition and mood. Clinical Endocrinology.

[16] Campos GM, Mazzini GS, Altieri MS, Docimo S, DeMaria EJ, Rogers AM (2021). ASMBS position statement on the rationale for performance of upper gastrointestinal endoscopy before and after metabolic and bariatric surgery. Surg Obes Relat Dis.

[17] Campos JM, Mello FST de, Ferraz ÁAB, Brito JN de, Nassif PAN, Galvão-Neto M dos P (2012). Dilatação endoscópica de anastomose gastrojejunal após bypass gástrico. arq bras cir dig.

[18] de Quadros LG, Galvão MDP, Campos JM, Kaiser RL, Grecco E, Flamini M (2017). Validation of a new method for the endoscopic measurement of post-bariatric gastric outlet using a standard guidewire: an observer agreement study. BMC Res Notes.

[19] Hales CM, Carroll MD, Fryar CD, Ogden CL (2020). Prevalence of Obesity and Severe Obesity Among Adults: United States, 2017-2018. NCHS Data Brief.

[20] Heymsfield SB, Wadden TA (2017). Mechanisms, Pathophysiology, and Management of Obesity. N Engl J Med.

[21] LeBlanc ES, Patnode CD, Webber EM, Redmond N, Rushkin M, O’Connor EA (2018). Behavioral and Pharmacotherapy Weight Loss Interventions to Prevent Obesity-Related Morbidity and Mortality in Adults: Updated Evidence Report and Systematic Review for the US Preventive Services Task Force. JAMA.

[22] Baretta GAP, Alhinho HCAW, Matias JEF, Marchesini JB, de Lima JHF, Empinotti C (2015). Argon plasma coagulation of gastrojejunal anastomosis for weight regain after gastric bypass. Obes Surg.

[23] Storm AC, Thompson CC (2017). Endoscopic Treatments Following Bariatric Surgery. Gastrointest Endosc Clin N Am.

[24] Di Lorenzo N, Antoniou SA, Batterham RL, Busetto L, Godoroja D, Iossa A (2020). Clinical practice guidelines of the European Association for Endoscopic Surgery (EAES) on bariatric surgery: update 2020 endorsed by IFSO-EC, EASO and ESPCOP. Surg Endosc.

[25] Peterson RM, Scott JD (2019). Managing Complications of Bariatric Surgery. Adv Surg.

[26] King WC, Hinerman AS, Belle SH, Wahed AS, Courcoulas AP (2018). Comparison of the Performance of Common Measures of Weight Regain After Bariatric Surgery for Association With Clinical Outcomes. JAMA.

[27] Bulajic M, Vadalà di Prampero SF, Boškoski I, Costamagna G (2021). Endoscopic therapy of weight regain after bariatric surgery. World J Gastrointest Surg.

[28] Kiani AK, Naureen Z, Pheby D, Henehan G, Brown R, Sieving P (2022). Methodology for clinical research. J Prev Med Hyg.

[29] Yimcharoen P, Heneghan HM, Singh M, Brethauer S, Schauer P, Rogula T (2011). Endoscopic findings and outcomes of revisional procedures for patients with weight recidivism after gastric bypass. Surg Endosc.

